# Corpus callosum dysgenesis causes novel patterns of structural and functional brain connectivity

**DOI:** 10.1093/braincomms/fcab057

**Published:** 2021-05-14

**Authors:** Diego Szczupak, Marina Kossmann Ferraz, Lucas Gemal, Patricia S Oliveira-Szejnfeld, Myriam Monteiro, Ivanei Bramati, Fernando R Vargas, IRC5 Consortium, Roberto Lent, Afonso C Silva, Fernanda Tovar-Moll

**Affiliations:** 1 Department of Neurobiology, University of Pittsburgh Brain Institute, University of Pittsburgh, Pittsburgh, PA 15261, USA; 2 D’Or Institute of Research and Education (IDOR), Rio de Janeiro 22281-100, Brazil; 3 Genetics and Molecular Biology Department, Federal University of the State of Rio de Janeiro, Rio de Janeiro 20270-330, Brazil; 4 Birth Defects Epidemiology Laboratory, Oswaldo Cruz Institute, Oswaldo Cruz Foundation, Rio de Janeiro 21040-360, Brazil; 5 Institute of Biomedical Sciences, Federal University of Rio de Janeiro, Rio de Janeiro 21941-902, Brazil

**Keywords:** corpus callosum, dMRI, fMRI, connectome, connectivity

## Abstract

Developmental malformations (dysgenesis) of the corpus callosum lead to neurological conditions with a broad range of clinical presentations. Investigating the altered brain connectivity patterns is crucial to understanding both adaptive and maladaptive neuroplasticity in corpus callosum dysgenesis patients. Here, we acquired structural diffusion-weighted and resting-state functional MRI data from a cohort of 11 corpus callosum dysgenesis patients (five with agenesis and six with hypoplasia) and compared their structural and functional connectivity patterns to healthy subjects selected from the Human Connectome Project. We found that these patients have fewer structural inter- and intra-hemispheric brain connections relative to healthy controls. Interestingly, the patients with callosal agenesis have a scant number of inter-hemispheric connections but manage to maintain the full integrity of functional connectivity between the same cortical regions as the healthy subjects. On the other hand, the hypoplasic group presented abnormal structural and functional connectivity patterns relative to healthy controls while maintaining the same total amount of functional connections. These results demonstrate that acallosal patients can compensate for having fewer structural brain connections and present functional adaptation. However, hypoplasics present atypical structural connections to different brain regions, leading to entirely new and abnormal functional brain connectivity patterns.

## Teaser

Szczupak et al. report that patients born with developmental corpus callosum malformations have abnormal structural brain connections. While acallosal patients have a scant number of inter-hemispheric connections, they preserve functional integrity and have a better prognosis than hypoplasic patients, which have atypical structural connections leading to abnormal functional connectivity patterns.

## Graphical Abstract

**Graphical Abstract:**
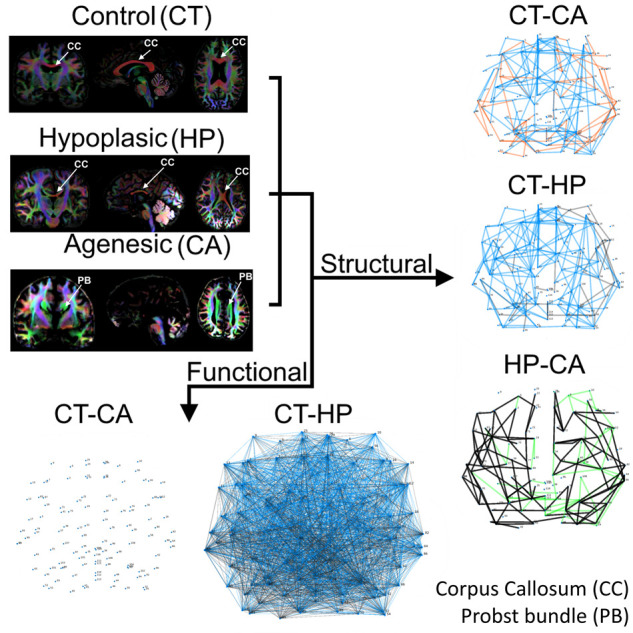

## Introduction

Developmental malformations (dysgenesis) of the corpus callosum (CCD) lead to neurological conditions with a broad range of clinical presentations, from entirely asymptomatic to severely handicapped subjects.^1^ In addition, CCD subjects often fit into the autism spectrum.^1^^,^^2^ When not linked to embryonically lethal causes, CCD usually falls into one of three different phenotypes: complete dysgenesis, also known as agenesis (CA); partial dysgenesis, which preserves a small remnant of the corpus callosum (CC; usually the genu); and hypoplasia (HP), with an anatomically typical but shortened CC.^1^^,^^3^ Although previous studies described the heterogeneous anatomical phenotypes of CCD patients and their neuropsychological presentations, the relationship between the altered structural brain wiring in CCD and the subsequent adaptive and maladaptive functional connectivity changes is still a subject of active research.^3^^,^^4^

The graph theory approach^5^^,^^6^ is an attractive way to investigate the changes in whole-brain connectivity in CCD patients. Previous studies used diffusion-weighted imaging (DWI) and resting-state functional MRI (rsfMRI) to access structural and functional changes in brain connectivity,^7^^,^^8^ but these studies did not make pairwise comparisons between the structural and functional network properties across the different CCD phenotypes.

In the present work, we use graph theory to investigate the relationship between structural (DWI) and functional (rsfMRI) data acquired in CCD patients. Our results demonstrate that the structural connectivity in both CA and HP subjects is different from that of healthy subjects. Surprisingly, although CA subjects can functionally connect the same brain regions compared to healthy controls, HP subjects connect different regions of the cortex, yielding to new patterns of global functional connectivity. These results point to a reorganization of structural brain connectivity in HP subjects, leading to abnormal and incoherent functional connectivity patterns that may explain some of the behavioural symptoms presented specifically by those subjects.

## Materials and methods

We performed a full MRI scan battery consisting of anatomical T1- and T2-weighted images, advanced DWI, and resting-state functional images (rs-fMRI) in 11 CCD patients (six with callosal agenesis and five with HP) to investigate the effect of different types of CCD in both structural and functional brain connectivity. Data from 45 healthy control subjects were analysed for comparisons with CCD data.

### CCD patients

**Figure 1:**
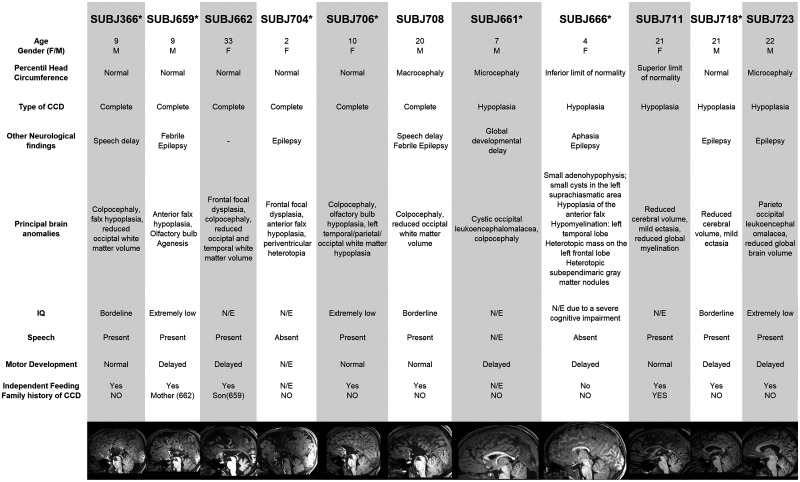
**Clinical/radiological demographic summary of the CCD patients.** Patients’ codes are noted on top. Asterisks denote that the patient was sedated during the MRI sessions. The bottom row shows a sagittal T1-weighted MRI of the patients’ brains. N/E, not examined.

We recruited 11 CCD patients in Rio de Janeiro, Brazil, for this study. The patients, aged 4–33 years (mean = 14.36, SD = 9.57), presented two different CCD phenotypes: six patients (three males, three females) had complete agenesis, and the other five (three males, two females) had callosal HP. Before enrolment in this study, the patients had a whole-brain anatomical MRI exam for diagnostic confirmation by experienced neuroradiologists (see Supplementary Fig. 1 for a detailed patient radiological summary panel). The Ethics Committee of our institution approved all procedures, and we obtained written informed consent from the patients or their parents. Figure 1 summarizes the patients’ clinical and radiological diagnoses. The team performed the clinical and morphological evaluation and interviewed the patients and their families to access speech, motor development, and independent feeding. Wechsler scales for Children and Adults^9^^,^^10^ were used to estimate intelligence quotient (Fig. 1).

#### Morphological abnormalities

**Figure 2:**
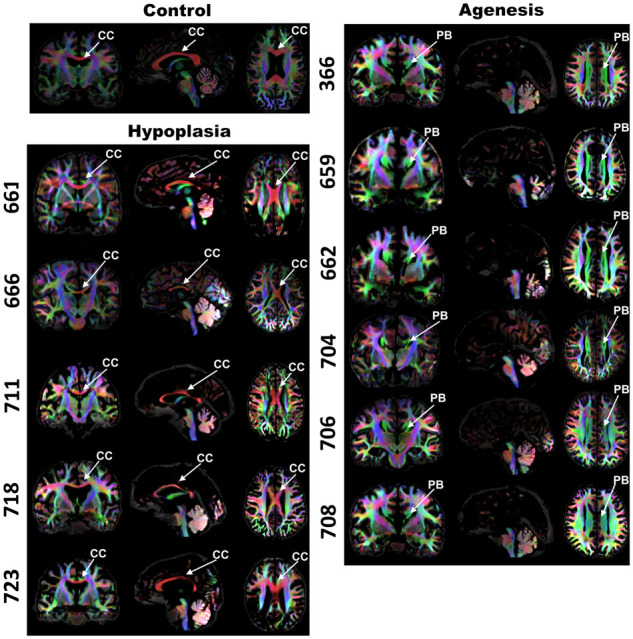
**White matter fibre orientation distribution maps for CCD patients.** The rows show the case number and three orthogonal anatomical planes (left to right: coronal, sagittal, and axial) for each respective patient. The top row shows a healthy control subject. Green represents the anteroposterior axis, red depicts the mediolateral axis, and blue denotes the dorsoventral axis. CC=corpus callosum; PB=probst bundle.

Our patients appraise two different CCD phenotypes. The first is HP, which is characterized, in a sagittal view, by a smaller but anatomically identified CC.^4^ The size reduction can occur either along the anteroposterior axis (e.g. Fig. 2, Subject 666) or the dorsoventral axis (e.g. Fig. 2, Subject 661). In this group, we can also see morphological abnormalities that indicate white matter reorganization. The coronal view of Subject 666 (Fig. 2) illustrates an excellent example of a patient with a hypoplasic CC where the main component of the fibre orientation distribution is not only laterolateral (red) but also anteroposterior (green). This abnormal pattern points to an asymmetrical white matter reorganization that can also be seen in the axial view of the same subject, indicating that these individuals might have other white matter defects outside of the CC.

The second patient group comprises individuals who entirely lack the CC (callosal agenesis, CA). All of them have a robust anatomical alteration called the Probst Bundle.^3^^,^^4^^,^^11^

### MRI acquisition

All patients were submitted to a single MRI session to undergo a T1-weighted anatomical image sequence, one diffusion-weighted image sequence, and one resting-state functional sequence. Images have undergone quality assessment regarding motion artefacts and signal–noise ratio. In specific cases of particularly young or handicapped subjects who experienced extreme discomfort in laying in the scanner, they were administered sevoflurane inhalation anaesthesia.

#### T1-weighted MRI sequence

For each CCD patient, we performed a whole-brain T1-weighted 3D MRI sequence in a 3T Magnetom Prisma Scanner (Siemens Healthineers), using the following pulse sequence parameters: repetition time [TR]/echo time [TE]/inversion time [TI] = 2400/2.2/1000 ms; field of view = 208× 300×320 mm^3^; matrix size = 260×375×400; spatial resolution = 0.8×0.8×0.8 mm^3^. These image datasets were sent to an experienced neuroradiologist to confirm the CCD diagnosis (Fig. 1).

### Diffusion-weighted image

We acquired all subject images following the Human Connectome Project criteria for 3.0 Tesla machines. The diffusion scheme of 200 directions was split into two different shells with *b* values of 1500 and 3000 s/mm^2^ with a 1.5-mm isotropic resolution acquired in two different phase encoding directions to minimize drop-out signal. CCD subjects were compared with 45 healthy control subjects (14 males, 31 females) from the Human Connectome Project retest database^12^ to allow an unbiased comparison. For this purpose, we subsampled the Human Connectome Project data to 1.5 mm and made a composite of the images, as shown in Fig. 2. We could not match the healthy subjects’ ages to those of the patients because the range of ages was too broad. However, we believe this limitation had minimal impact on our study since we were only looking for brain connections that are well developed at birth,^13^ and not for tissue properties such as myelination proxies that change with the maturation of these connections.^14^

DWI images were corrected for eddy-currents and geometric distortions and denoised. Furthermore, we estimated a response function using the Dholander algorithm and calculated the fibre orientation distribution in Mrtrix.^15^

### rs-fMRI

We obtained rs-fMRI data from the same CCD patients and healthy controls as in the anatomical study. The rs-fMRI images followed the Human Connectome Project protocol with an isotropic spatial resolution of 2 mm. Because we acquired rs-fMRI data from the patients with only one phase encoding direction, we processed the healthy control subjects in the same manner to allow an unbiased comparison. The data were preprocessed with Analysis of Functional NeuroImages (AFNI) software to spatially registered the time points in subject space, performed the despike correction, regressed motion derived parameters, and temporally filtered the time series with a band-pass filter from 0.1 to 0.01 Hz in AFNI software, following their automated and established pipeline.^16^

### Connectome construction

#### DWI connectome

We used Mrtrix to calculate the tractogram by generating tracts targeting the whole brain from seeds with the following parameters: cut-off = 0.06 (stops the tractography when fibre orientation distribution value is lower than 0.06), select 10 M (selecting 10 million streamlines to assure enough coverage of the tractogram), maximal length 250 (to ensure that the streamlines are not longer than the brain length). After creating the tractogram, we performed a global intensity correction using the Mrtrix command *tcksift*. Then, we registered the AAL 116 ROIs human cortical atlas^17^ to each subject and used the command *tck2connectome* to calculate the connectome adjacency matrix.

#### Rs-fMRI connectome

To generate the connectome adjacency matrices, we used the Matlab automated toolbox GRETNA^18^ to calculate the ROI-to-ROI functional connectivity with an absolute correlation value threshold of 0.1 using the AAL 116 ROIs atlas.

### Analysis

#### Base matrix construction

We created the matrices’ binary by thresholding each edge value by 50 streamlines for DWI and using a minimum absolute correlation coefficient of 0.1 for the rs-fMRI datasets. A base matrix for each CCD group—agenesis (CA), HP, and healthy controls—was created as defined by Szczupak et al.^19^ The rationale is that the base matrix computes only the common edges to all subjects, allowing us to identify common patterns to the CCD phenotypes, addressing the limitations of small group sizes and large range of age within groups.

#### Edges quantification

We quantified the number of non-zero elements of the base matrices and compared them with the adjacency matrices. These values corresponded to the number of connections in the brains and were divided into three groups: total connections, inter-hemispheric connections, and intra-hemispheric connections. We also added a fourth metric, the ratio of inter- to intra-hemispheric connections representing a balance in brain connectivity. We compared these metrics between CCD groups with an ANOVA test with Tukey multiple comparisons correction post-test by comparing all groups against all groups.

#### Network-based statistics

We calculated the Small-worldliness, assortativity, hierarchy, and efficiency with network-based-statistics parameters in GRETNA and compared these parameters between CCD groups with ANOVA with a multiple comparison correction by comparing all CCD conditions. Small worldliness is a metric that represents small world motifs in a given network. These motifs are defined as a number of small local connections and few long-distance connections and are well associated with an efficient way of transmitting the information. Efficiency is defined as the number of different paths connecting two nodes and relates not only to the efficiency but also to the network’s redundancy. Hierarchy, on the other hand, relates to the classification of the individual nodes (ROI) as measured by the degree (number of connections) of each node, and assortativity if these nodes communicate with nodes of similar class, relating to the pattern and type of connectivity of the network.^5^^,^^6^ These specific metrics can describe not only if there is a loss in function of a network (small worldliness and efficiency), but if the network has undergone an altered development (hierarchy and assortativity).

### Data availability

The MRI datasets generated and analysed for this study can be requested from the corresponding author and follow the D’Or Institute of Research and Education and institutional IRB guidelines.

## Results

### DWI network-based statistics

**Figure 3:**
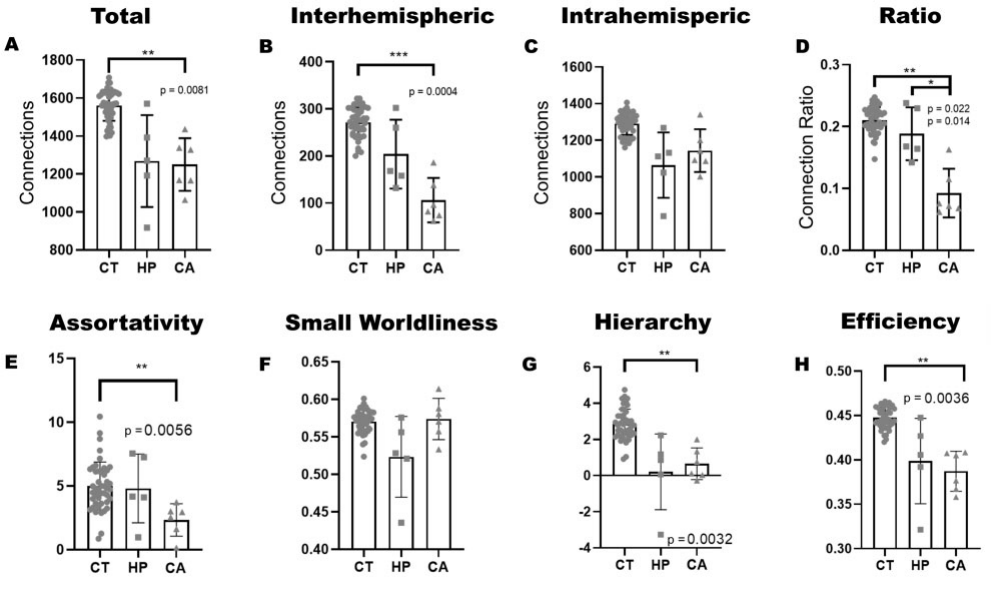
**DWI features.** Comparison of the number of edges in all CCD conditions (**A–D**) and Network-Based-Statistics (**E–H**). Multiple comparisons were performed with one-way ANOVA with Tukey multiple comparison post-test, and the *P* values of significant differences are shown in the graphs. CT=control; CA=callosal agenesis; HP=hypoplasia; intra=intra-hemispheric; inter=inter-hemispheric.

We first quantified each individual’s number of edges in the DWI connectomes to investigate the CCD subjects’ connectivity patterns relative to controls. The CA group’s total number of edges is significantly smaller than the controls (Fig. 3A). This difference is explained by the reduced number of inter-hemispheric connections (Fig. 3B) but not the intra-hemispheric ones (Fig. 3C), which led to a connection shift from inter- to intra-hemispheric (Fig. 3D). This difference in edge quantity is also reflected in the network properties of Small Worldliness (Fig. 3E), Efficiency (Fig. 3F), Hierarchy (Fig. 3G), and Assortativity (Fig. 3H).

The lack of statistical differences relating to the hypoplasic patients is probably due to this group’s larger intrinsic variability, as their large variance can be observed in network properties (Fig. 3) and anatomical phenotype (Fig. 2).

### DWI base matrix

**Figure 4:**
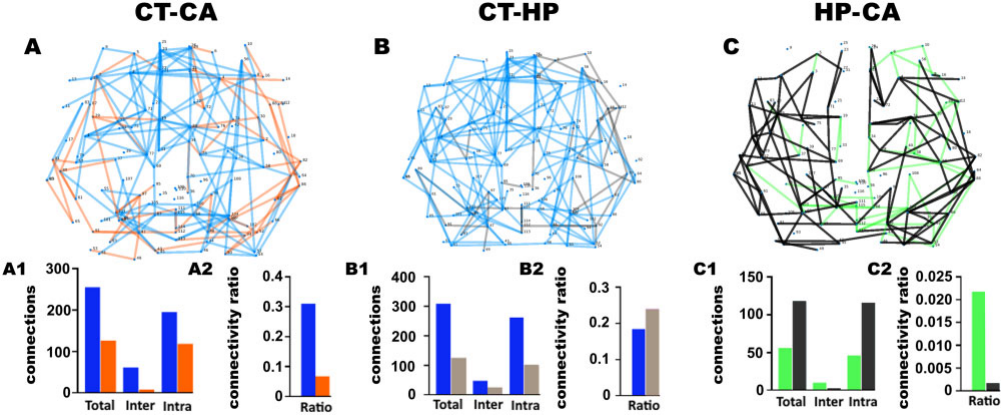
**Display and quantification of the DWI base matrices comparison.** Graphs display axial views of the brain with lines depicting the edges. Blue edges represent missing connections, and orange and grey edges indicate aberrant connections in every individual of CA and HP subjects, respectively. The graphs in (**A**) and (**B**) show the comparison of callosal agenesis (CA) and hypoplasic (HP) patients with healthy controls (CT), respectively. The graph in (**C**) shows the comparison between the two different CCD phenotypes. Green shows the connections present only in HP and black only in CA. The bar plots below show the quantification of the number of edges. CT=controls; CA=corpus callosum agenesis; HP=hypoplasia; intra=intra-hemispheric; inter=inter-hemispheric.

We then compared what was common to all of the subjects to visualize a group pattern that was previously hidden by the individual variance, a structural backbone. Figure 4 shows pairwise comparisons of the DWI base matrix (see Materials and methods) between the CCD patients and the typical subjects. Connectomic graph shows an axial view of the brain with lines depicting in blue the missing connections (that are typically present in control subjects but not in CCD patients) and in orange (CA) and grey (HP) the aberrant connections (that normally do not form in control subjects, but are present in every CCD patient). Figure 4C compares the two different types of CCD to show phenotype-specific patterns (green representing HP-specific connections and black CA-specific connections).

The comparison of controls and callosal agenesis (CA) patients (Fig. 4A) show that the CA group has a total number of missing connections higher than the aberrant connections, especially the inter-hemispheric but also the intra-hemispheric connections due to their large number of aberrant connections (Fig. 4A1). Nevertheless, we can still see an overall shift from inter-hemispheric to intra-hemispheric connectivity (Fig. 4A2) in CA subjects.

In comparison with CA, the hypoplasia group (HP) has more missing connections (blue) and less aberrant connections (grey). Both inter- and intra-hemispheric connections are reduced in HP patients (Fig. 4B1). Unlike the CA patients, we could not see a shift from inter- to intra-hemispheric connectivity in HP patients (Fig. 4B2).

The difference between CCD phenotypes can be clearly seen in Fig. 4C, where the HP group has less total and less intra-hemispheric connections relative to CA (Fig. 4C1). Although both CCD groups have a small number of inter-hemispheric connections, the HP group has a higher amount connecting posterior cortical regions (Fig. 4C) than the CA group (Fig. 4C1). The inter-hemispheric ratio is much higher in the HP group relative to CA patients (Fig. 4C2).

### rs-fMRI network-based statistics

**Figure 5:**
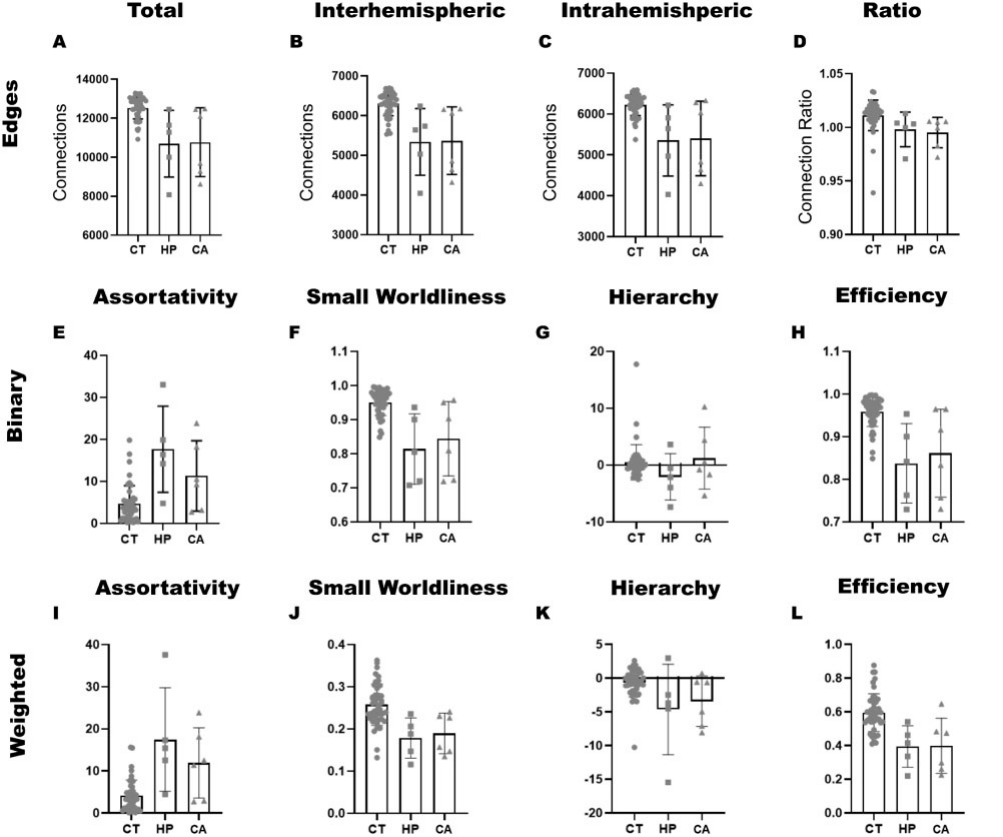
**Resting-state fMRI features.** Quantification of the number of connections in the brain (**A–D**). Network-based-statistics of the binary rs-fMRI connectomes (**E–H**) and the weighted by connectivity strength (**I–L**). Multiple comparisons were performed with one-way ANOVA with Tukey multiple comparison post-test. No statistical significance was found. CT=controls; HP=hypoplasic; CA=agenesis.

To evaluate the functional network properties of the CCD patients, we performed the same analysis of the rs-fMRI data for the structural connectome and included the networks weighted by the resting-state connectivity strength (see Materials and methods). We found no functional differences between groups when comparing CCD patients and control subjects regarding the number of edges or the network-based-statistics and between the CCD phenotypes (Fig. 5). We also considered the anaesthesia effect and tested to see if it was a confounding factor, but we found no difference in the awake patients (Supplementary Fig. 2).

### rs-fMRI base matrix

**Figure 6:**
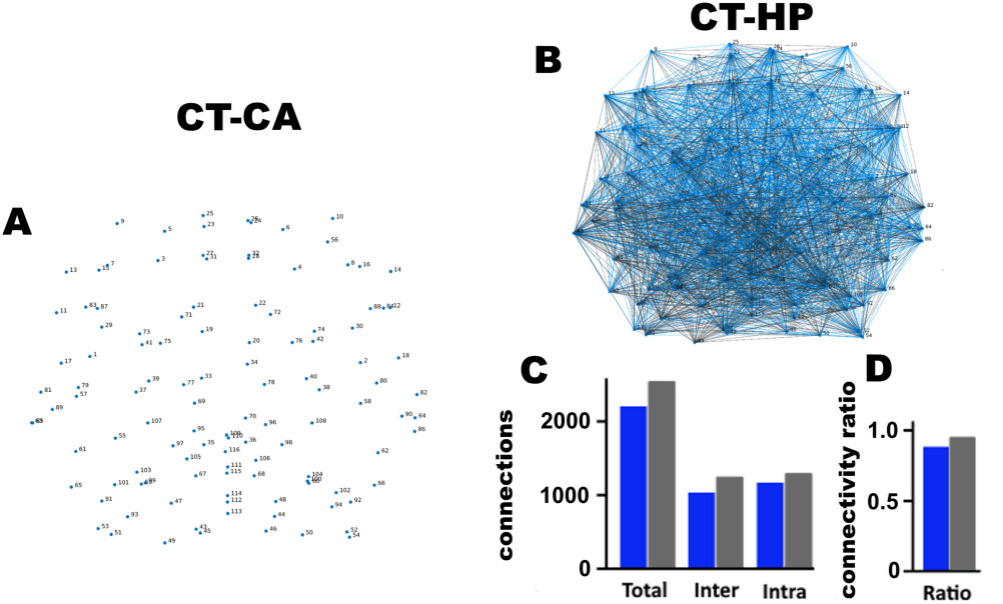
**rs-fMRI base comparison.** Display and quantification of the base matrices comparison. Graphs display axial views of the brain with lines depicting the edges. Blue edges represent missing connections, and magenta edges denote aberrant connections. (**A**) shows a graph of the comparison of controls (CT) with agenesis (CA). No quantification of edges is shown since the group difference was null. (**B**) shows graphs of the comparison of controls (CT) with hypoplasia (HP), respectively. (**C**) shows the quantification of the number of edges, and (**D**) shows the ratio. CT=control; AC=agenesis; HP=hypoplasia; intra=intra-hemispheric; inter=inter-hemispheric.

To investigate whether individual connectivity profiles influenced any functional patterns, we analysed the rs-fMRI backbone in the same manner as the DWI connectome (see Materials and methods). In this analysis, we compared what is common to all subjects to find the ultimate common pattern of that particular group. The connectomic graph shows an axial view of the brain with lines depicting only abnormal connections. The blue colour shows the missing connections typically present in controls, and the grey colour represents the aberrant connections that normally do not form in controls (Fig. 6).

We first compared the control group with the CA group and found that CA patients connect precisely the same brain regions as healthy subjects despite their complete absence of the CC (Fig. 6A). This result shows that CA patients can maintain global functional connectivity integrity and suggest their functional adaptation to the complete lack of a corpus callosum.

We then compared the controls with the hypoplasics and found strikingly different results (Fig. 6B). The HP group had a similar number of total missing connections (blue) and aberrant connections (magenta) (Fig. 6C). This pattern was the same in the inter-hemispheric connections (Fig. 6C) and intra-hemispheric connections (Fig. 6C), yielding no shift of connectivity (Fig. 6D). These sets of results show that although having a similar number of edges, the HP group completely misconnects the regions of the brain.

## Discussion

It is challenging to investigate the consequences of brain malformations to brain function thoroughly. Due to the diverse anatomical CCD presentations in the brain, patients develop a broad clinical spectrum that ranges from being utterly inconsequential to normal function. On one end, to being severely debilitating and requiring assisted care, on the other extreme. Understanding the causes of this diversity, especially the relationship between the pathological anatomical and functional connectivity in CCD, is crucial to therapeutical approaches development.

The multiple, heterogeneous malformations of the brain also make it challenging to group patients according to a single criterium, such as the complete absence versus the partial presence of the corpus callosum, as the interindividual variability of phenotypic presentations within the same group may limit the interpretation of group-based data analysis. To adequately address this limitation, we chose to analyse the data in two complementary ways.

First, we performed a traditional, group-based analysis that clearly shows that CA patients, but not HP, differ from healthy subjects in the global network properties of structural connectivity (Fig. 3). While it is easy to accept the anatomical divergence of CA patients, it is somewhat surprising that HP patients did not differ from healthy controls in their structural connectivity. Furthermore, neither patient cohort differed from healthy controls in their functional connectivity properties (Fig. 5).

Second, we also performed a different type of analysis that computes only the similarities between subjects in each group but not the differences. This novel approach allowed us to verify that both CA and HP patients differ structurally from controls (Fig. 4), but that somehow the acallosals can functionally connect the same regions as the controls, while the hypoplasics have the same number of brain connections, albeit connecting different brain regions (Fig. 6).

These findings collectively suggest that while CA patients undergo a significant structural rearrangement of their brains, they can fully compensate functionally for their lack of the CC. Meanwhile, the HP group is disorganized both anatomically and functionally. Their anatomical disorganization leads to significant functional deficits that make them more vulnerable to CCD, which is in agreement with the prognosis described previously.^20^

### Anatomical variability

In the patient cohorts studied here, all subjects of the CA group presented the Probst bundle, which is a longitudinal tract, presumed to be formed by misguided callosal axons that fail to cross the midline and fasciculate with pre-existing longitudinal bundles (as the cingulate bundle). The Probst bundle presence is a common anatomical feature of CCD.^3^^,^^4^^,^^11^ At least at a macroscopic, anatomical level, the brains of our acallosal cohort seemed to be very homogeneous, not showing any clear white matter missing bundles other than the callosal absence.

In contrast, the hypoplasic group did not present (macroscopically) the Probst bundle, which might be overshadowed by the strong signal of the CC fibres in the DWI MRI. It is also noticeable that the shape, length, and width of the CC varies greatly between patients, suggesting that this group will naturally have a more substantial variance in connectivity metrics and might include subpopulations within the group. The higher variance of CCD connectivity indicators is in agreement with previous works^7^ that, although not separated by CCD type, show a larger spread of the distribution.

The small size (∼1.5 mm isotropic voxel) of the sagittal area of the other commissures, such as the posterior and the anterior, creates a partial volume effect and makes it challenging to arrive at a precise quantification. However, our previous work shows the existence of abnormal bundles through these commissures.^21^

The intrinsic variability of brain malformation subjects is a challenging aspect of investigating neural networks where the overall individualities obscure the patterns. To overcome this challenge, we have analysed the networks with a global approach, together with an approach that only considers what is common to all patients.

### Abnormal brain connectivity

#### Structural connectivity

We compared CA patients with those with callosal HP, using a recently proposed DWI method^12^ with superior *in vivo* resolution (1.5 mm isotropic) and a multishell diffusion scheme with two diffusion spheres. This approach allowed us to model the fibre orientation distributions and achieve a better detection of brain fibres^22^ to construct a structural connectome and compare it with an rs-fMRI brain networks connectome of the same subjects.

Our traditional network-based-statistics results show that CA patients have fewer connections than typical subjects, while the HP group was not different from healthy controls. This result can be explained by the total lack of the CC in CA cases and the more diverse connections that result from the morphological variance of the hypoplasic CC. In support of our findings, Yuan et al.^23^ recently showed that CCD patients have weaker inter-hemispheric and increased intra-hemispheric connectivity strength.

The base matrix approach was used to analyse only the patterns common to all subjects in each group and revealed that CA patients display a lower number of connections as a whole, as well as fewer inter- and intra-hemispheric connections. This difference can be divided into missing normal connections and aberrant connections. Overall, the CA has more missing typical than aberrant connections, which suggests that despite compensatory plasticity, it could not make up for the number of lost connections. HP subjects also show a loss of connections in both intra- and inter-hemispheric connectivity. Unlike the acallosals, the hypoplasics show a balance on the overall ratio of missing and abnormal connections. These results suggest that they equally lose intra- and inter-hemispheric connections as they generate aberrant intra- and inter-hemispheric alternatives.

The CCD phenotype difference reveals that CA subjects have a larger overall intra-hemispheric connectivity, while the HP has a larger (posterior) inter-hemispheric connectivity. These different connectivity patterns show how different types of CCD have their unique fingerprints, although this particular comparison does not show if these connections are aberrant or normal. The reason why HP produces a lower total number of connections may also be explained by the large intrinsic variance of this group that was removed in this analysis.

It is interesting to notice that most of the altered inter-hemispheric connections in CCD patients are heterotopic (Fig. 4), showing that CCD changes the balance between homotopic and heterotopic connections via the CC. The presence of heterotopic connections in CCD patients is a topic of growing interest in the literature.^4^^,^^24^

#### Functional connectivity

The traditional approach for the analysis of functional connectivity showed no significant differences between groups. These results may mean that by direct or indirect connections, the CCD patients can use different plastic mechanisms to connect the same number of brain regions and therefore compensate for the partial or complete lack of the corpus callosum.

Using the base matrix approach to reveal the common circuitry, we found that the functional resting-state backbone connectome of the agenesis group connects precisely the same regions as the control group. These results suggest that somehow the inter-hemispheric structural connections of these patients could transfer the information to the other hemisphere and connect the same structures. It is well-known that CCD displays abnormal bundles^3^^,^^4^^,^^11^ and that some of them can connect the hemispheres and transfer information between them,^21^ but the present connectomic study indicates that multiple pathways may maintain normal brain connectivity.

This does not necessarily mean that these connections’ coherence, speed, and accuracy are necessarily the same as in typical subjects. As this is a binary observation and does not reflect the weight of the connection, the axons might innervate the same brain region but not necessarily participate in the same intra-region circuits. Nevertheless, it illustrates the brain’s adaptive strength, capable of connecting both hemispheres even without any callosal remnant.

However, when we compare the backbone of the functional connectivity in the control group with the hypoplasic group, we can see that both have a similar number of inter- and intra-hemispheric connections. These patterns show that the hypoplasic group connects abnormally the brain regions. These results add up to the observation that fibres crossing through the hypoplasic CC may not do it in an orderly manner and possibly connect brain regions different from controls. These abnormal connections may cause dysfunction, being a cause of symptoms experienced by these patients. These findings are in agreement with the literature that points to HP patients being more affected by CCD during development.^20^ A recent work investigated the relationship of connectivity metrics and clinical outcome in CCD patients.^25^ These results are in agreement with our findings for the AC group, in which the maintenance of intra-hemispheric structural connections leads to normotypical functional network patterns.

### Global network properties

The structural DWI networks revealed that agenesis subjects have lower small worldliness, global efficiency, hierarchy and assortativity. These findings suggest that the structural networks impact the inter-hemispheric crossing and limit overall brain connectivity. It is important to the point that not only the inter-hemispheric connections were altered in the CA but also the intra-hemispheric connections comprised a large portion of the altered connections. Therefore, the changes in network-based-statistics are a combination of the effects of altered intra- and inter-hemispheric connectivity.

The hypoplasic group presents with wide variability, including some patients similar to the healthy controls and others more similar to the agenesic group. This suggests that there could be different subtypes of hypoplasic patients, although the sample size was perhaps too small to assure this conclusion.

In contrast, the resting-state connectome showed no significant differences between groups, suggesting that the network of these patients, although abnormal, still presents similar properties and therefore has similar levels of complexity (Fig. 5).

These results show that the abnormal networks are not randomly generated, suggesting still unknown compensatory cellular mechanisms during development that would produce the same properties as the control group brain networks. And they also point out that the study of network properties without the anatomical placement of these connections might obscure important biological differences.

A pioneering study^26^ found similar results by investigating an agenesic cohort connectome, revealing that multiple brain regions change node degrees. Although the work did not check if these connections were inter- or intra-hemispheric, it showed that the absence of the CC produces changes throughout the cortex. More recently, the same group investigated the DWI connectome in prenatal brains.^8^ Although with lower resolution, it was possible to show how these brains can vary and add to the evidence that the CC malformations generate overall brain changes.

The present results are also compatible with a previous rs-fMRI connectome study^7^ that investigated CCD patients (without separating them by subtypes) and showed that bilateral networks could be found in the patients despite the absence of a CC. The same results have previously been obtained by Tyszka et al.,^27^ showing no differences in network-based-statistics, although focal nodal interactions were changed.

Finally, two possible limitations of our work relate to the small group size and wide age variation of the patient cohorts. We are actively recruiting additional patients on an ongoing basis to allow us to formally address the impact of these limitations in future studies.

## Supplementary material

Supplementary material is available at *Brain Communications* online.

## Supplementary Material

fcab057_Supplementary_Data

## Abbreviations

CA =: callosum agenesis
CC =: corpus callosum
CCD =: corpus callosum dysgenesis
DWI =: diffusion-weighted imaging
HP =: hypoplasia
MRI =: magnestic resonance imaging
rs-FMRI =: resting-state functional MRI
E =: echo time
TR =: repetition time
