# Supplementary material for: Corpus callosum dysgenesis causes novel patterns of structural and functional brain connectivity
Source: Brain Commun. 2021 May 14;3(2):fcab057. doi: 10.1093/braincomms/fcab057 (PMC8152904; doi:10.1093/braincomms/fcab057)
Supplement: fcab057_Supplementary_Data [file fcab057_supplementary_data.pdf]

Supplementary Material

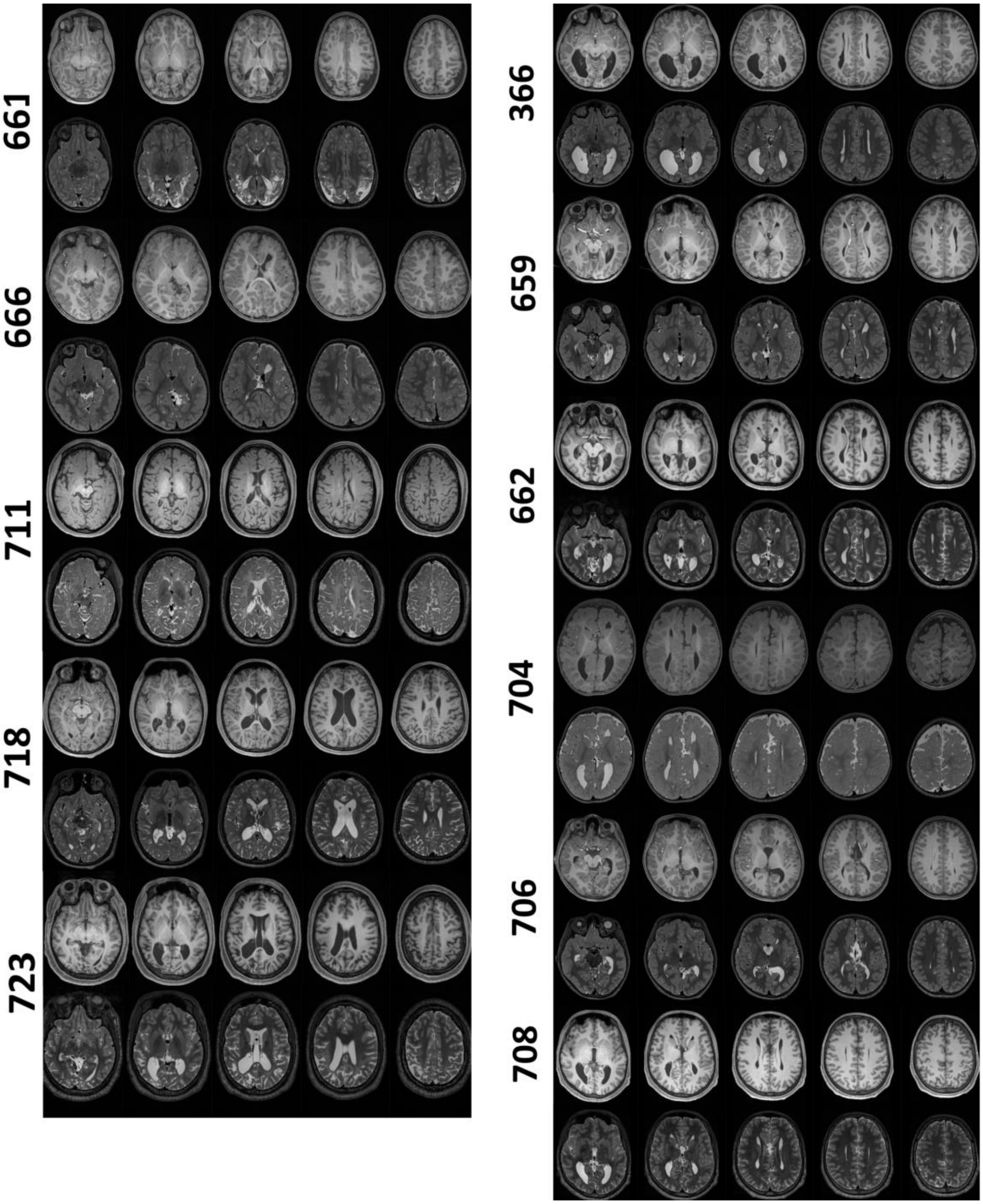

Supplementary Figure 1. Anatomical panel. Axial slices of CCD subjects. For each subject is presented a T1 image (first row) and a T2 image (second row). Slices were chosen to cover the whole cortex and allow the full visualization of brain abnormalities.

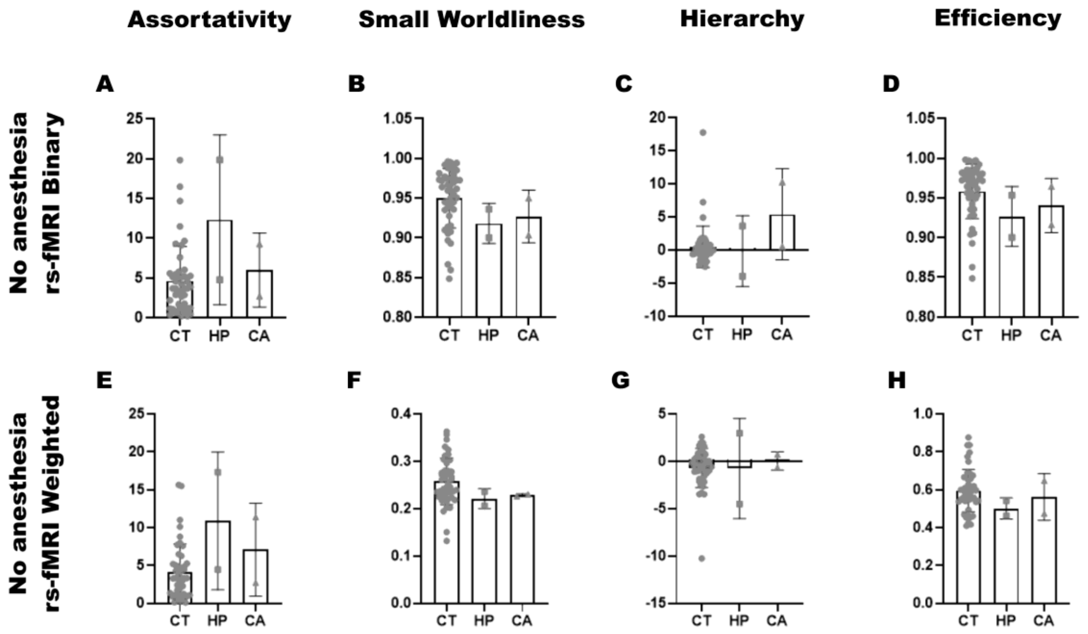

Supplementary Figure 2. NBS without anesthesia. Quantification of NBS in all groups of the patients that had no anesthesia in the rsFMRI binary and weighted by functional connectivity strength. CT = Controls, CA = Agenesis, and HP = Hypoplasia.

Radiological subject description

Subject 366

Complete CC absence. Normal cranial and cerebral volume (subjective evaluation), agenesis of the anterior and inferior falx. Lateral ventricles present a classic colpocephaly, dilated and asymmetric atrium, temporal, and occipital horns. Irregular ependyma surface (no heterotopias). Absent inter-thalamic adherence. Reduced

bilateral occipital and temporal white matter with normal intensity. Retrocerebellar meningeal cyst.

Subject 659

Complete CC absence. Normal cranial and cerebral volume (subjective evaluation), agenesis of the anterior and inferior falx. The lateral ventricle presents atypical colpocephaly (completely parallel ventricles with deviated frontal horns, sharp-angled borders, narrow temporal horns, and dilated occipital horns). Irregular ependyma surface (no heterotopias). Absent inter-thalamic adherence. Normal white matter volume and intensity. Olfactory bulb agenesis.

Subject 662

Complete CC absence. Normal cranial and cerebral volume (subjective evaluation), agenesis of the anterior and inferior falx. The lateral ventricle presents classic colpocephaly. Deviated ventricles' frontal horn with ectasia, sharp-angled borders, and narrow temporal horns, and dilated occipital horns). Irregular ependyma surface (no heterotopias). Absent inter-thalamic adherence. Bilateral reduced mesial temporal and occipital white matter volume with normal intensity. Mesial right frontal cortical dysplasia. Anterior vermis and olfactory bulb hypoplasia.

Subject 704

Complete CC absence. Normal cranial and cerebral volume (subjective evaluation), agenesis of the anterior and inferior falx. The lateral ventricle presents parallel

colpocephaly, deviated ventricles' frontal horn with ectasia, sharp-angled borders, and narrow temporal horns, and dilated occipital horns). Irregular ependyma surface. Bilateral mesial temporal and occipital reduced white matter. Left frontal lobe white matter hypomyelination. Associated with cortical dysplasia and focal heterotopia. Periventricular heterotopias. Small Blake cyst.

Subject 706

Complete CC absence. Normal cranial and cerebral volume (subjective evaluation), agenesis of the anterior and inferior falx. Lateral ventricles were presenting "buffalo horn"-like shaped colpocephaly, thin and concave frontal horns with parallel symmetric bodies, atrium and temporal horn ectasia, occipital horn choroid fissure. Absent thalamic adherence. Reduced left parietal, temporal, and occipital mesial white matter volume. Olfactory bulb hypoplasia.

Subject 708

Complete CC absence. Normal cranial and cerebral volume (subjective evaluation), agenesis of the anterior and inferior falx. Lateral ventricles present a classic colpocephaly. Absent inter-thalamic adherence. Reduced mesial temporal and occipital white matter volume with normal intensity.

Subject 661

Hypoplasic CC. Microcephalic cranium. Severe occipital/temporal leukoencephalomalacia. Frontal white matter thinning with normal intensity. Anterior falx dysplasia. Colpocephaly. Arachnoid cyst in the cisterna magna.

Subject 666

Hypoplasic CC. Normal cranium (subjective). Left frontal lobe reduction with normal intensity white matter thinning. Frontal lobe cortical dysplasia with an inferior thickening. Focal heterotopias. Anterior falx dysplasia. Lateral ventricles present colpocephaly, asymmetric lateral ventricles, frontal horn with ectasia, sharp-angled borders, and dilated occipital horns. Bilateral arachnoid cyst.

Subject 711

Hypoplasic CC. Microcephalic cranium (subjective). Anterior falx dysplasia. Colpocephaly. Lateral ventricles present ectasia. Diffuse hypomyelination. Anterior vermis hypoplasia.

Subject 718

Hypoplasic CC. Microcephalic cranium (subjective). Anterior falx dysplasia. Colpocephaly. Lateral ventricles' present diffuse ectasia. Anterior vermis hypoplasia. Reduced deep white matter volume. Atrophy of the cerebellum hemispheres.

Subject 723

Hypoplasic CC. Reduced cerebral volume (subjective) due to posterior leukoencephalomalacia. Moderate diffuse bilateral asymmetric ectasia. Reduced bilateral occipital white matter volume. Reduced cerebellar hemisphere volume.
